# 9-(2-Chloro­phen­yl)-4a-hy­droxy-3,4,4a,5,6,7,9,9a-octa­hydro-2*H*-xanthene-1,8-dione

**DOI:** 10.1107/S1600536814005297

**Published:** 2014-03-15

**Authors:** Qiu-Ling Liu, Xin-Yan Wu, Feng Gao, Dan Bao, Fang-Ming Wang

**Affiliations:** a Jiangsu University of Science and Technology, Zhenjiang 212003, People’s Republic of China

## Abstract

In the title compound, C_19_H_19_ClO_4_, the di­hydro­pyran ring and the cyclo­hexane ring adopt a half-chair conformation and a chair conformation, respectively. The cyclo­hexene ring has an envelope conformation with the central CH_2_ C atom as the flap. This atom is disordered over two positions [site-occupancy ratio = 0.744 (12):0.256 (12)] above and below the mean plane formed by the other five atoms. In the crystal, O—H⋯O hydrogen bonds between hy­droxy and carbonyl groups link mol­ecules into chains propagating along [001].

## Related literature   

For the background, synthesis and activities of xanthenes, see: Knight & Stephens (1989 ▶); Srividya *et al.* (1996 ▶); Menchen *et al.* (2003 ▶); Reddy *et al.* (2009 ▶); Mehdi *et al.* (2011 ▶); Altieri *et al.*(2013 ▶). For related structures, see: Hua *et al.* (2006 ▶); Yang *et al.* (2011 ▶).
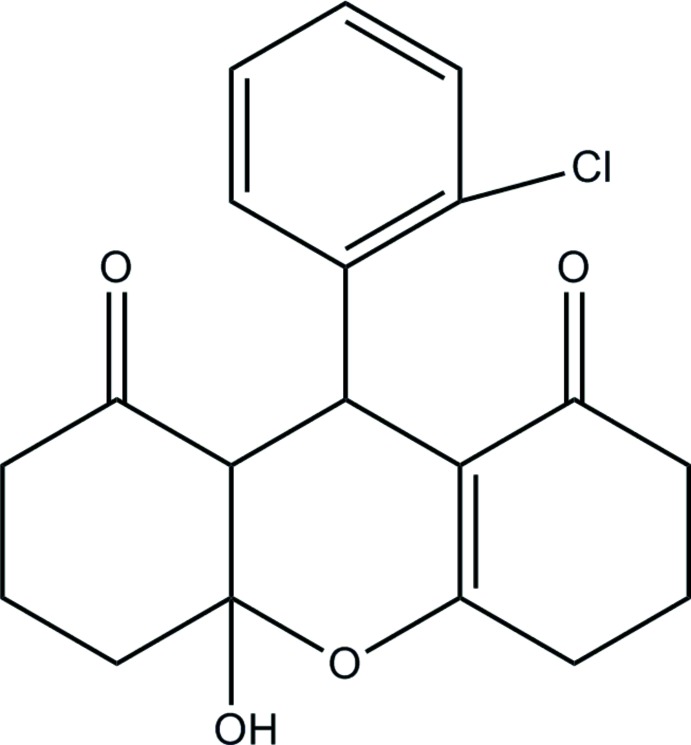


## Experimental   

### 

#### Crystal data  

C_19_H_19_ClO_4_
*M* *_r_* = 346.79
Monoclinic, 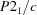

*a* = 15.3099 (13) Å
*b* = 9.2815 (8) Å
*c* = 12.3216 (11) Å
β = 110.716 (1)°
*V* = 1637.7 (2) Å^3^
*Z* = 4
Mo *K*α radiation
μ = 0.25 mm^−1^
*T* = 291 K
0.25 × 0.23 × 0.18 mm

#### Data collection  

Bruker SMART APEXII CCD area-detector diffractometer
Absorption correction: multi-scan (*SADABS*; Bruker, 2001 ▶) *T*_min_ = 0.94, *T*_max_ = 0.96
12319 measured reflections
3207 independent reflections
2208 reflections with *I* > 2σ(*I*)
*R*_int_ = 0.037

#### Refinement  

*R*[*F* ^2^ > 2σ(*F* ^2^)] = 0.042
*wR*(*F* ^2^) = 0.137
*S* = 1.00
3207 reflections
227 parameters
H-atom parameters constrained
Δρ_max_ = 0.24 e Å^−3^
Δρ_min_ = −0.24 e Å^−3^

### 

Data collection: *APEX2* (Bruker, 2007 ▶); cell refinement: *SAINT* (Bruker, 2007 ▶); data reduction: *SAINT*; program(s) used to solve structure: *SHELXTL* (Sheldrick, 2008 ▶); program(s) used to refine structure: *SHELXTL*; molecular graphics: *SHELXTL*; software used to prepare material for publication: *SHELXTL*.

## Supplementary Material

Crystal structure: contains datablock(s) I, New_Global_Publ_Block. DOI: 10.1107/S1600536814005297/xu5775sup1.cif

Structure factors: contains datablock(s) I. DOI: 10.1107/S1600536814005297/xu5775Isup2.hkl

Supporting information file. DOI: 10.1107/S1600536814005297/xu5775Isup3.cml

CCDC reference: 990638

Additional supporting information:  crystallographic information; 3D view; checkCIF report

## Figures and Tables

**Table 1:** Hydrogen-bond geometry (Å, °)

*D*—H⋯*A*	*D*—H	H⋯*A*	*D*⋯*A*	*D*—H⋯*A*
O4—H4*O*⋯O2^i^	0.82	2.00	2.784 (2)	161

Symmetry code: (i) 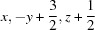.

**Fig. 1.**
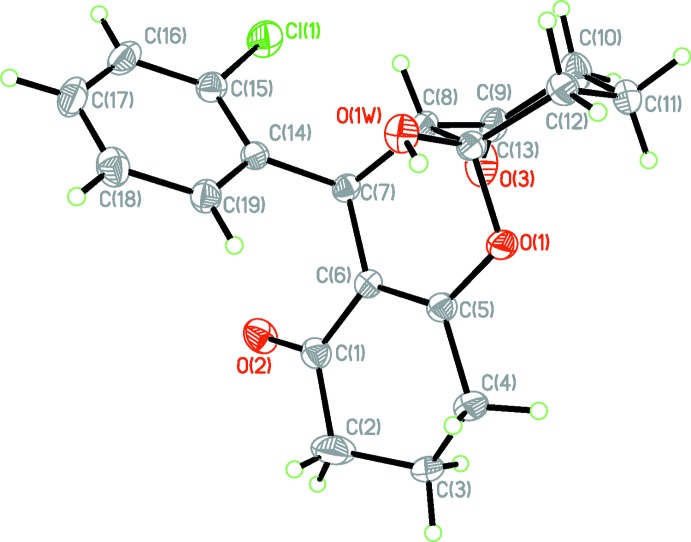
The structure of the title compound with labeling and displacement ellipsoids drawn at the 30% probability level.

**Fig. 2.**
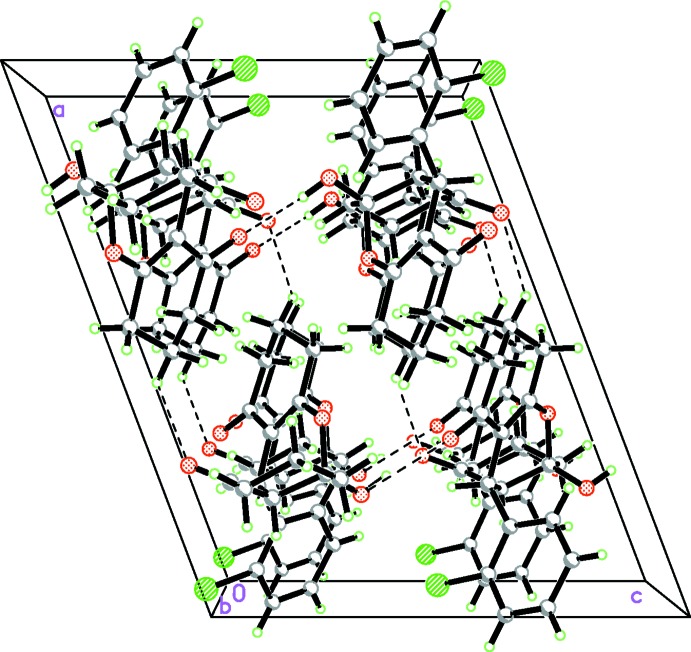
The packing diagram of title compound as viewed along b axis (dotted lines are H-bonds)

## supplementary crystallographic information

### 1. Introduction

Xanthenes and its derivatives are an important class of organic compounds with biological and pharmacological activity, such as anti­tumoral, fungicidal, anti-inflammatory, bactericidal properties, and so on. (Knight & Stephens, 1989; Srividya *et al.*, 1996; Menchen *et al.*, 2003; Reddy *et al.*, 2009; Mehdi *et al.*, 2011). In addition, xanthene derivatives are essential synthetic inter­mediates in heterocyclic compounds synthesis (Altieri *et al.*, 2013).

In the main structure of this compound (Figure 1), there exist central di­hydro­pyran ring (O1/C5/C6/C7/C8/C13) adopts a half-chair conformations while the cyclo­hexane ring adopts a chair conformation. The cyclo­hexene ring displays an approximate envelope conformation, and in the crystal this ring is disordered over two positions in an occupancy ratio of 0.744 (12):0.256 (12) with the flap atom located on the opposite sides of the mean plane formed by other five atoms. The bond lengths and angles are comparable to those in a related structure (Hua *et al.*, 2006; Yang *et al.*, 2011). The crystal packing is stabilized by inter­molecular O—H···O hydrogen bond between hydroxyl and carbonyl groups, which links the molecules into supra­molecular chains running along the c-axis direction.

### 2. Experimental

^1^H NMR (400 MHz, DMSO-*d*_6_) *δ*: 7.11-7.31 (m, 4H), 6.93(s, 1H), 4.63(s, 1H), 3.06(s, 1H), 2.39(m, 3H), 2.21(m, 4H), 2.02(m, 2H), 1.85(m, 2H),1.58(m, 1H); IR (KBr pellet): 3442(br), 2962(m), 1711(m), 1640(m), 1609(s), 1389(s), 1079(s) ,773(m); MS (ESI) m/z : 347.1 (M—H^+^).

### 2.1. Synthesis and crystallization

To a 100 mL flask, there were added 1,3-cyclo­hexane­dione (4.5 g, 40mmol) and 2-chloro-benzaldehyde (2.8 g, 20mmol)in 40mL methanol, using L-proline (0.5 g) as the catalyst. The mixture were stirred at room temperature for 8h. The white precipitate was vacuum filtered and washed with methanol. And Then dried under vacuum to yield the white solid (6.1 g, 88% yield).

Single crystals of the title compound suitable for X-ray structure determination were obtained by slow evaporation from mixture solution of anhydrous ethanol and di­chloro­methane (v: v = 1:1) at room temperature to yield colorless, block-shaped crystal.

### 2.2. Refinement

All H atoms were fixed geometrically and treated as riding with C—H = 0.97 Å (methyl­ene), 0.98 Å (methine), 0.93 Å (phenyl), O–H = 0.82 Å (hy­droxy), with individual displacement parameters as *U*iso(H) = 1.2*U*eq (C) or 1.2*U*eq (O) .

### Crystal data

**Table:**

C_19_H_19_ClO_4_	*F*(000) = 728
*M**_r_* = 346.79	*D*_x_ = 1.406 Mg m^−^^3^
Monoclinic, *P*2_1_/*c*	Mo *K*α radiation, λ = 0.71073 Å
Hall symbol: -P 2ybc	Cell parameters from 2557 reflections
*a* = 15.3099 (13) Å	θ = 2.6–22.6°
*b* = 9.2815 (8) Å	µ = 0.25 mm^−^^1^
*c* = 12.3216 (11) Å	*T* = 291 K
β = 110.716 (1)°	Block, colorless
*V* = 1637.7 (2) Å^3^	0.25 × 0.23 × 0.18 mm
*Z* = 4	

### Data collection

**Table:**

Bruker SMART APEXII CCD area-detector diffractometer	3207 independent reflections
Radiation source: sealed tube	2208 reflections with *I* > 2σ(*I*)
Graphite monochromator	*R*_int_ = 0.037
φ and ω scans	θ_max_ = 26.0°, θ_min_ = 1.4°
Absorption correction: multi-scan (*SADABS*; Bruker, 2001)	*h* = −18→18
*T*_min_ = 0.94, *T*_max_ = 0.96	*k* = −11→11
12319 measured reflections	*l* = −15→15

### Refinement

**Table:**

Refinement on *F*^2^	Primary atom site location: structure-invariant direct methods
Least-squares matrix: full	Secondary atom site location: difference Fourier map
*R*[*F*^2^ > 2σ(*F*^2^)] = 0.042	Hydrogen site location: inferred from neighbouring sites
*wR*(*F*^2^) = 0.137	H-atom parameters constrained
*S* = 1.00	*w* = 1/[σ^2^(*F*_o_^2^) + (0.0852*P*)^2^] where *P* = (*F*_o_^2^ + 2*F*_c_^2^)/3
3207 reflections	(Δ/σ)_max_ < 0.001
227 parameters	Δρ_max_ = 0.24 e Å^−^^3^
0 restraints	Δρ_min_ = −0.24 e Å^−^^3^

### Special details

**Table:**

Geometry. All e.s.d.'s (except the e.s.d. in the dihedral angle between two l.s. planes) are estimated using the full covariance matrix. The cell e.s.d.'s are taken into account individually in the estimation of e.s.d.'s in distances, angles and torsion angles; correlations between e.s.d.'s in cell parameters are only used when they are defined by crystal symmetry. An approximate (isotropic) treatment of cell e.s.d.'s is used for estimating e.s.d.'s involving l.s. planes.
Refinement. Refinement of *F*^2^ against ALL reflections. The weighted *R*-factor *wR* and goodness of fit *S* are based on *F*^2^, conventional *R*-factors *R* are based on *F*, with *F* set to zero for negative *F*^2^. The threshold expression of *F*^2^ > σ(*F*^2^) is used only for calculating *R*-factors(gt) *etc*. and is not relevant to the choice of reflections for refinement. *R*-factors based on *F*^2^ are statistically about twice as large as those based on *F*, and *R*- factors based on ALL data will be even larger.

### Fractional atomic coordinates and isotropic or equivalent isotropic displacement parameters (Å^2^)

**Table:**

	*x*	*y*	*z*	*U*_iso_*/*U*_eq_	Occ. (<1)
C1	0.36292 (16)	0.6212 (3)	0.22858 (19)	0.0374 (5)	
C2	0.4586 (2)	0.5704 (3)	0.3045 (3)	0.0679 (9)	
H2A	0.4519	0.5038	0.3618	0.082*	
H2B	0.4865	0.5178	0.2569	0.082*	
C3	0.5220 (3)	0.6854 (6)	0.3646 (6)	0.0557 (18)	0.744 (12)
H3A	0.5405	0.7363	0.3075	0.067*	0.744 (12)
H3B	0.5778	0.6441	0.4211	0.067*	0.744 (12)
C3'	0.4986 (10)	0.6331 (16)	0.4163 (11)	0.048 (4)	0.256 (12)
H3'A	0.5649	0.6240	0.4362	0.058*	0.256 (12)
H3'B	0.4820	0.5808	0.4733	0.058*	0.256 (12)
C4	0.48086 (16)	0.7929 (3)	0.4227 (2)	0.0474 (7)	
H4A	0.5185	0.8798	0.4380	0.057*	
H4B	0.4826	0.7543	0.4966	0.057*	
C5	0.38240 (15)	0.8304 (2)	0.35106 (17)	0.0325 (5)	
C6	0.32588 (14)	0.7506 (2)	0.26163 (17)	0.0304 (5)	
C7	0.22743 (14)	0.7965 (2)	0.19183 (17)	0.0294 (5)	
H7A	0.2224	0.7950	0.1103	0.035*	
C8	0.21218 (14)	0.9542 (2)	0.21988 (16)	0.0287 (5)	
H8A	0.1447	0.9677	0.1986	0.034*	
C9	0.24489 (16)	1.0626 (3)	0.14999 (18)	0.0368 (5)	
C10	0.2394 (2)	1.2177 (3)	0.1799 (2)	0.0475 (6)	
H10A	0.1745	1.2456	0.1591	0.057*	
H10B	0.2671	1.2769	0.1357	0.057*	
C11	0.29051 (17)	1.2435 (3)	0.3093 (2)	0.0408 (6)	
H11A	0.3568	1.2265	0.3284	0.049*	
H11B	0.2822	1.3429	0.3278	0.049*	
C12	0.25337 (16)	1.1442 (3)	0.38055 (19)	0.0374 (6)	
H12A	0.2898	1.1578	0.4621	0.045*	
H12B	0.1893	1.1705	0.3685	0.045*	
C13	0.25672 (14)	0.9869 (2)	0.35002 (17)	0.0296 (5)	
C14	0.15332 (15)	0.6935 (2)	0.20126 (18)	0.0308 (5)	
C15	0.06507 (16)	0.6870 (2)	0.11488 (19)	0.0364 (5)	
C16	−0.00219 (17)	0.5888 (3)	0.1178 (2)	0.0461 (6)	
H16A	−0.0601	0.5866	0.0582	0.055*	
C17	0.01752 (18)	0.4942 (3)	0.2097 (2)	0.0491 (7)	
H17A	−0.0268	0.4268	0.2119	0.059*	
C18	0.10288 (18)	0.5000 (3)	0.2982 (2)	0.0450 (6)	
H18A	0.1157	0.4378	0.3611	0.054*	
C19	0.17003 (16)	0.5983 (2)	0.29409 (19)	0.0364 (5)	
H19A	0.2275	0.6007	0.3545	0.044*	
Cl1	0.03711 (4)	0.80536 (8)	−0.00262 (5)	0.0547 (2)	
O1	0.35645 (10)	0.95441 (16)	0.38789 (12)	0.0361 (4)	
O2	0.31864 (12)	0.55190 (18)	0.14146 (13)	0.0472 (5)	
O3	0.27329 (14)	1.0251 (2)	0.07442 (15)	0.0588 (5)	
O4	0.21588 (11)	0.89704 (16)	0.40837 (12)	0.0387 (4)	
H4O	0.2496	0.8920	0.4769	0.058*	

### Atomic displacement parameters (Å^2^)

**Table:**

	*U*^11^	*U*^22^	*U*^33^	*U*^12^	*U*^13^	*U*^23^
C1	0.0396 (13)	0.0346 (13)	0.0383 (12)	0.0004 (10)	0.0139 (10)	−0.0021 (10)
C2	0.0515 (18)	0.0584 (19)	0.078 (2)	0.0237 (15)	0.0032 (15)	−0.0164 (16)
C3	0.028 (2)	0.049 (3)	0.082 (4)	0.0068 (18)	0.009 (2)	−0.012 (3)
C3'	0.036 (7)	0.058 (8)	0.040 (6)	0.010 (6)	0.001 (5)	0.010 (5)
C4	0.0312 (13)	0.0437 (16)	0.0537 (14)	0.0042 (11)	−0.0020 (11)	−0.0068 (12)
C5	0.0302 (12)	0.0337 (13)	0.0322 (11)	0.0010 (10)	0.0093 (9)	−0.0022 (9)
C6	0.0281 (11)	0.0311 (12)	0.0308 (10)	−0.0014 (9)	0.0089 (9)	−0.0009 (9)
C7	0.0275 (11)	0.0328 (12)	0.0254 (10)	−0.0014 (9)	0.0064 (8)	−0.0030 (8)
C8	0.0258 (11)	0.0289 (11)	0.0292 (10)	−0.0023 (9)	0.0070 (8)	−0.0001 (9)
C9	0.0373 (13)	0.0400 (14)	0.0280 (11)	−0.0077 (10)	0.0052 (9)	0.0007 (9)
C10	0.0578 (16)	0.0362 (14)	0.0457 (14)	−0.0037 (12)	0.0149 (12)	0.0069 (11)
C11	0.0434 (14)	0.0301 (13)	0.0463 (13)	−0.0036 (11)	0.0129 (11)	−0.0038 (10)
C12	0.0381 (13)	0.0355 (13)	0.0388 (12)	0.0011 (10)	0.0138 (10)	−0.0054 (10)
C13	0.0281 (11)	0.0308 (12)	0.0298 (10)	−0.0006 (9)	0.0101 (9)	−0.0010 (9)
C14	0.0290 (11)	0.0291 (12)	0.0334 (10)	−0.0005 (9)	0.0098 (9)	−0.0080 (9)
C15	0.0334 (12)	0.0380 (14)	0.0353 (11)	−0.0004 (10)	0.0092 (9)	−0.0054 (10)
C16	0.0298 (13)	0.0542 (17)	0.0524 (14)	−0.0094 (12)	0.0123 (11)	−0.0158 (13)
C17	0.0439 (15)	0.0463 (16)	0.0654 (17)	−0.0161 (12)	0.0298 (13)	−0.0139 (13)
C18	0.0543 (16)	0.0380 (15)	0.0505 (14)	−0.0036 (12)	0.0281 (12)	−0.0005 (11)
C19	0.0358 (12)	0.0347 (13)	0.0381 (12)	−0.0038 (10)	0.0122 (10)	−0.0025 (10)
Cl1	0.0444 (4)	0.0587 (5)	0.0442 (4)	−0.0027 (3)	−0.0052 (3)	0.0053 (3)
O1	0.0286 (8)	0.0362 (9)	0.0366 (8)	0.0016 (7)	0.0031 (6)	−0.0094 (7)
O2	0.0585 (11)	0.0397 (10)	0.0387 (9)	0.0032 (8)	0.0114 (8)	−0.0113 (8)
O3	0.0818 (14)	0.0590 (12)	0.0456 (10)	−0.0228 (11)	0.0348 (10)	−0.0083 (9)
O4	0.0442 (9)	0.0397 (10)	0.0322 (8)	−0.0043 (8)	0.0136 (7)	0.0019 (7)

### Geometric parameters (Å, º)

**Table:**

C1—O2	1.229 (3)	C8—H8A	0.9800
C1—C6	1.447 (3)	C9—O3	1.210 (3)
C1—C2	1.509 (3)	C9—C10	1.495 (3)
C2—C3'	1.419 (11)	C10—C11	1.526 (3)
C2—C3	1.456 (5)	C10—H10A	0.9700
C2—H2A	0.9700	C10—H10B	0.9700
C2—H2B	0.9700	C11—C12	1.515 (3)
C3—C4	1.490 (5)	C11—H11A	0.9700
C3—H3A	0.9700	C11—H11B	0.9700
C3—H3B	0.9700	C12—C13	1.513 (3)
C3—H3'A	1.0608	C12—H12A	0.9700
C3'—C4	1.514 (13)	C12—H12B	0.9700
C3'—H3B	1.1976	C13—O4	1.386 (2)
C3'—H3'A	0.9601	C13—O1	1.461 (2)
C3'—H3'B	0.9599	C14—C15	1.395 (3)
C4—C5	1.494 (3)	C14—C19	1.396 (3)
C4—H4A	0.9700	C15—C16	1.385 (3)
C4—H4B	0.9700	C15—Cl1	1.746 (2)
C5—O1	1.348 (3)	C16—C17	1.380 (4)
C5—C6	1.355 (3)	C16—H16A	0.9300
C6—C7	1.509 (3)	C17—C18	1.375 (4)
C7—C14	1.519 (3)	C17—H17A	0.9300
C7—C8	1.541 (3)	C18—C19	1.389 (3)
C7—H7A	0.9800	C18—H18A	0.9300
C8—C9	1.520 (3)	C19—H19A	0.9300
C8—C13	1.535 (3)	O4—H4O	0.8200
			
O2—C1—C6	122.0 (2)	C8—C7—H7A	106.3
O2—C1—C2	119.5 (2)	C9—C8—C13	110.33 (17)
C6—C1—C2	118.4 (2)	C9—C8—C7	113.29 (17)
C3'—C2—C3	39.2 (6)	C13—C8—C7	112.07 (17)
C3'—C2—C1	117.7 (5)	C9—C8—H8A	106.9
C3—C2—C1	114.3 (3)	C13—C8—H8A	106.9
C3'—C2—H2A	71.1	C7—C8—H8A	106.9
C3—C2—H2A	108.7	O3—C9—C10	122.2 (2)
C1—C2—H2A	108.7	O3—C9—C8	121.7 (2)
C3'—C2—H2B	131.5	C10—C9—C8	116.0 (2)
C3—C2—H2B	108.7	C9—C10—C11	110.9 (2)
C1—C2—H2B	108.7	C9—C10—H10A	109.5
H2A—C2—H2B	107.6	C11—C10—H10A	109.5
C2—C3—C4	114.6 (4)	C9—C10—H10B	109.5
C2—C3—H3A	107.5	C11—C10—H10B	109.5
C4—C3—H3A	107.7	H10A—C10—H10B	108.1
C2—C3—H3B	109.5	C12—C11—C10	110.56 (19)
C4—C3—H3B	109.6	C12—C11—H11A	109.5
H3A—C3—H3B	107.6	C10—C11—H11A	109.5
C2—C3—H3'A	97.7	C12—C11—H11B	109.5
C4—C3—H3'A	101.8	C10—C11—H11B	109.5
H3A—C3—H3'A	127.7	H11A—C11—H11B	108.1
H3B—C3—H3'A	20.2	C13—C12—C11	112.95 (18)
C2—C3'—C4	115.4 (8)	C13—C12—H12A	109.0
C2—C3'—H3B	99.5	C11—C12—H12A	109.0
C4—C3'—H3B	96.4	C13—C12—H12B	109.0
C2—C3'—H3'A	105.4	C11—C12—H12B	109.0
C4—C3'—H3'A	105.5	H12A—C12—H12B	107.8
H3B—C3'—H3'A	15.0	O4—C13—O1	108.80 (16)
C2—C3'—H3'B	111.6	O4—C13—C12	112.99 (17)
C4—C3'—H3'B	111.0	O1—C13—C12	104.07 (16)
H3B—C3'—H3'B	122.2	O4—C13—C8	107.92 (16)
H3'A—C3'—H3'B	107.3	O1—C13—C8	108.71 (16)
C3—C4—C5	112.5 (2)	C12—C13—C8	114.14 (18)
C3—C4—C3'	37.4 (5)	C15—C14—C19	116.4 (2)
C5—C4—C3'	111.1 (5)	C15—C14—C7	121.16 (19)
C3—C4—H4A	109.1	C19—C14—C7	122.39 (19)
C5—C4—H4A	109.1	C16—C15—C14	122.5 (2)
C3'—C4—H4A	136.0	C16—C15—Cl1	117.97 (18)
C3—C4—H4B	109.1	C14—C15—Cl1	119.50 (17)
C5—C4—H4B	109.1	C17—C16—C15	119.4 (2)
C3'—C4—H4B	75.0	C17—C16—H16A	120.3
H4A—C4—H4B	107.8	C15—C16—H16A	120.3
O1—C5—C6	124.11 (19)	C18—C17—C16	119.8 (2)
O1—C5—C4	110.88 (18)	C18—C17—H17A	120.1
C6—C5—C4	125.0 (2)	C16—C17—H17A	120.1
C5—C6—C1	118.8 (2)	C17—C18—C19	120.3 (2)
C5—C6—C7	122.1 (2)	C17—C18—H18A	119.9
C1—C6—C7	119.07 (18)	C19—C18—H18A	119.9
C6—C7—C14	113.68 (17)	C18—C19—C14	121.5 (2)
C6—C7—C8	109.90 (16)	C18—C19—H19A	119.2
C14—C7—C8	113.82 (17)	C14—C19—H19A	119.2
C6—C7—H7A	106.3	C5—O1—C13	117.90 (16)
C14—C7—H7A	106.3	C13—O4—H4O	109.5
			
O2—C1—C2—C3'	−166.3 (10)	C7—C8—C9—C10	−175.13 (18)
C6—C1—C2—C3'	13.3 (10)	O3—C9—C10—C11	−126.3 (2)
O2—C1—C2—C3	150.0 (4)	C8—C9—C10—C11	53.9 (3)
C6—C1—C2—C3	−30.3 (5)	C9—C10—C11—C12	−55.0 (3)
C3'—C2—C3—C4	−57.7 (7)	C10—C11—C12—C13	54.7 (3)
C1—C2—C3—C4	46.9 (7)	C11—C12—C13—O4	−175.08 (17)
C3—C2—C3'—C4	56.8 (10)	C11—C12—C13—O1	67.1 (2)
C1—C2—C3'—C4	−38.5 (17)	C11—C12—C13—C8	−51.3 (2)
C2—C3—C4—C5	−40.4 (7)	C9—C8—C13—O4	172.60 (17)
C2—C3—C4—C3'	55.3 (7)	C7—C8—C13—O4	−60.2 (2)
C2—C3'—C4—C3	−58.2 (10)	C9—C8—C13—O1	−69.5 (2)
C2—C3'—C4—C5	41.6 (15)	C7—C8—C13—O1	57.7 (2)
C3—C4—C5—O1	−162.2 (4)	C9—C8—C13—C12	46.1 (2)
C3'—C4—C5—O1	157.5 (8)	C7—C8—C13—C12	173.36 (17)
C3—C4—C5—C6	18.4 (5)	C6—C7—C14—C15	−156.35 (19)
C3'—C4—C5—C6	−22.0 (9)	C8—C7—C14—C15	76.8 (2)
O1—C5—C6—C1	178.4 (2)	C6—C7—C14—C19	21.9 (3)
C4—C5—C6—C1	−2.2 (3)	C8—C7—C14—C19	−105.0 (2)
O1—C5—C6—C7	1.6 (3)	C19—C14—C15—C16	−2.0 (3)
C4—C5—C6—C7	−179.0 (2)	C7—C14—C15—C16	176.3 (2)
O2—C1—C6—C5	−172.6 (2)	C19—C14—C15—Cl1	178.38 (16)
C2—C1—C6—C5	7.7 (3)	C7—C14—C15—Cl1	−3.3 (3)
O2—C1—C6—C7	4.3 (3)	C14—C15—C16—C17	0.7 (3)
C2—C1—C6—C7	−175.3 (2)	Cl1—C15—C16—C17	−179.66 (19)
C5—C6—C7—C14	−116.6 (2)	C15—C16—C17—C18	1.1 (4)
C1—C6—C7—C14	66.6 (2)	C16—C17—C18—C19	−1.5 (4)
C5—C6—C7—C8	12.3 (3)	C17—C18—C19—C14	0.1 (3)
C1—C6—C7—C8	−164.54 (18)	C15—C14—C19—C18	1.6 (3)
C6—C7—C8—C9	84.2 (2)	C7—C14—C19—C18	−176.7 (2)
C14—C7—C8—C9	−147.02 (17)	C6—C5—O1—C13	15.9 (3)
C6—C7—C8—C13	−41.5 (2)	C4—C5—O1—C13	−163.57 (18)
C14—C7—C8—C13	87.3 (2)	O4—C13—O1—C5	72.7 (2)
C13—C8—C9—O3	131.7 (2)	C12—C13—O1—C5	−166.63 (17)
C7—C8—C9—O3	5.1 (3)	C8—C13—O1—C5	−44.6 (2)
C13—C8—C9—C10	−48.6 (3)		

### Hydrogen-bond geometry (Å, º)

**Table:**

*D*—H···*A*	*D*—H	H···*A*	*D*···*A*	*D*—H···*A*
O4—H4*O*···O2^i^	0.82	2.00	2.784 (2)	161

Symmetry code: (i) *x*, −*y*+3/2, *z*+1/2.
